# Subcutaneous Emphysema, Pneumothorax and Pneumomediastinum Following Endoscopic Sphincterotomy

**DOI:** 10.4021/gr232w

**Published:** 2010-09-20

**Authors:** Leonardo L. Schiavon, Rodrigo A. Rodrigues, Frank S. Nakao, Veruska O. Di Sena, Angelo P. Ferrari, Ermelindo D. Libera

**Affiliations:** a Federal University of Sao Paulo, Gastroenterology Division, Hospital Sao Paulo, Sao Paulo, Brazil

**Keywords:** Subcutaneous emphysema, Pneumothorax, Pneumomediastinum, Sphincterotomy, Endoscopic retrograde cholangiopancreatography

## Abstract

Retroperitoneal perforation during therapeutic endoscopic retrograde cholangiopancreatography (ERCP) is uncommon and is usually manifested by abdominal pain, fever and leukocytosis. We report the case of a patient with post-ERCP subcutaneous emphysema, pneumomediastinum and pneumothorax treated conservatively. A 79-year-old woman with a diagnosis of choledocholitiasis was referred to our institution for an elective outpatient therapeutic ERCP. At the end of the procedure, subcutaneous emphysema was observed, and a thoracic computed tomography revealed a right pneumothorax and pneumomediastinum. Supportive care was instituted and she was discharged asymptomatic after 10 days of hospitalization. Subcutaneous emphysema, pneumothorax and pneumomediastinum are potencial complications of ERCP and sphincterotomy. We review the other cases previously reported and discuss the management.

## Introduction

Therapeutic endoscopic retrograde cholangiopancreatography (ERCP) is a well established method for treating recurrent or retained common bile duct stones. The reported rate of complications related to this procedure ranges from 0.8% to 45%, including pancreatitis, hemorrhage, perforation and cholangitis. The main risk factors for complication are difficult bile duct cannulation, cirrhosis, suspected sphincter of Oddi dysfunction, pre-cut technique and previous gastrointestinal surgery [1, 2]. Perforation during ERCP occurs in 2.1% of cases [3, 4]. Peritoneal perforation most frequently results from gastrointestinal wall injury during the procedure [2]. Retroperitoneal perforation is usually related to extensive sphincterotomy beyond the intramural portion of bile and pancreatic ducts. The classic features of retroperitoneal perforation include abdominal pain, fever and leukocytosis. However, most cases turn out well with minor clinical manifestations if an early diagnosis is made [5]. Subcutaneous emphysema, pneumomediastinum and pneumothorax as a result of retroperitoneal perforation after sphincterotomy are rarely reported complications [6-11]. We report the case of a patient with post-ERCP subcutaneous emphysema, pneumomediastinum and pneumothorax treated conservatively.

## Case Report

A 79-year-old woman had a recent episode of jaundice and abdominal pain that resolved spontaneously. A transabdominal ultrasound revealed a dilated common hepatic duct with stone inside and she was referred to an elective outpatient ERCP because of choledocholitiasis. Her past medical history included diabetes and a cholecystectomy for cholelithiasis 16 years before. Examination was unremarkable. Aspartate aminotransferase, alanine aminotransferase, alkaline phosphatase, and bilirubin were within the normal range.

**Figure 1:**
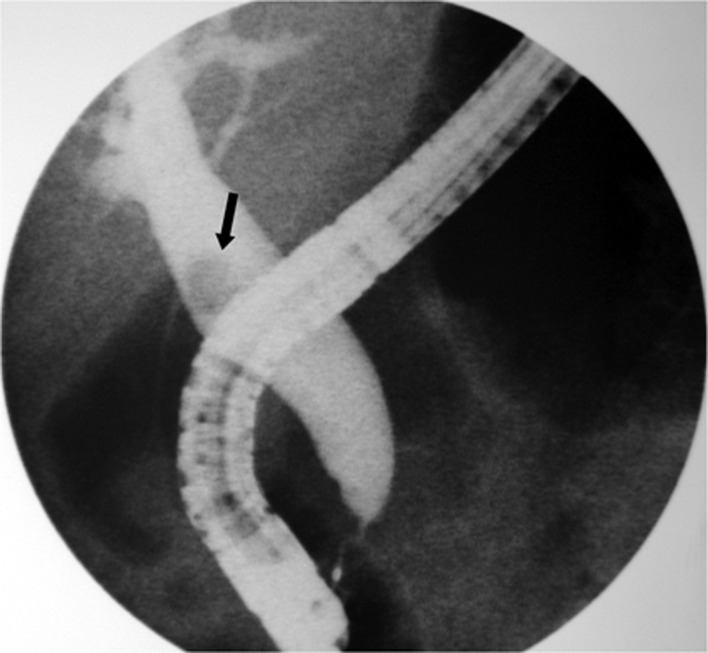
Cholangiography showing a dilatated common bile duct with a single stone (black arrow).

**Figure 2:**
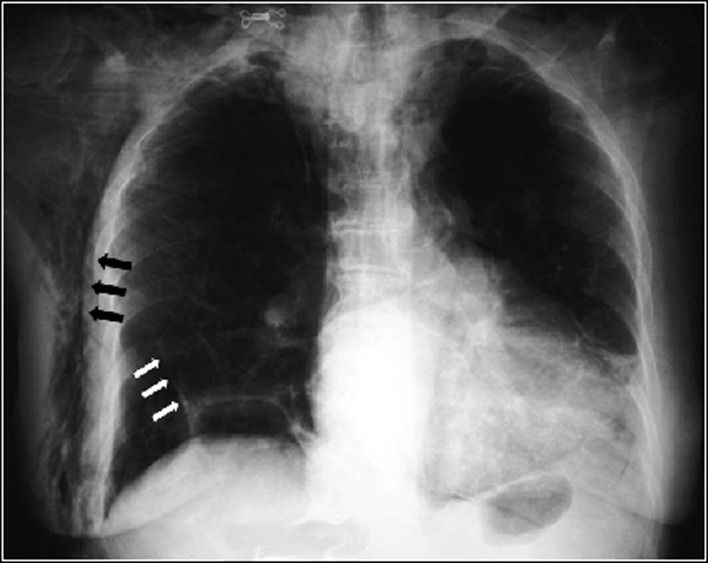
Chest x-ray showing diffuse subcutaneous emphysema (black arrows) and a right-side pneumothorax (white arrows).

**Figure 3:**
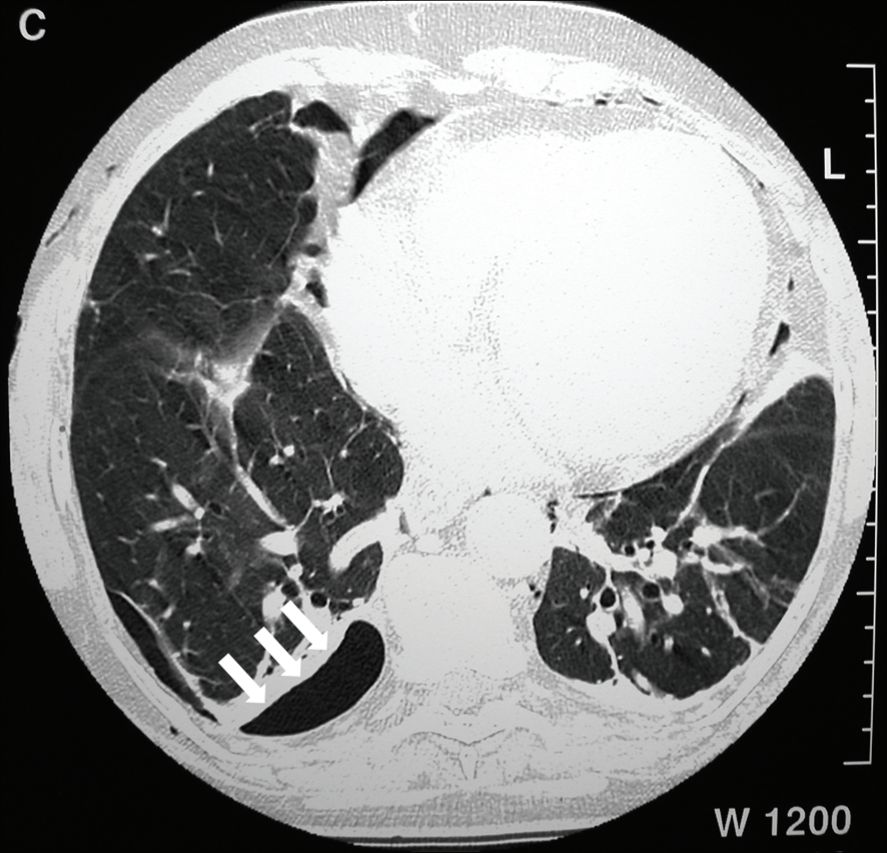
Thoracic computed tomography (CT) showing a small right-side pneumothorax (white arrows).

**Figure 4:**
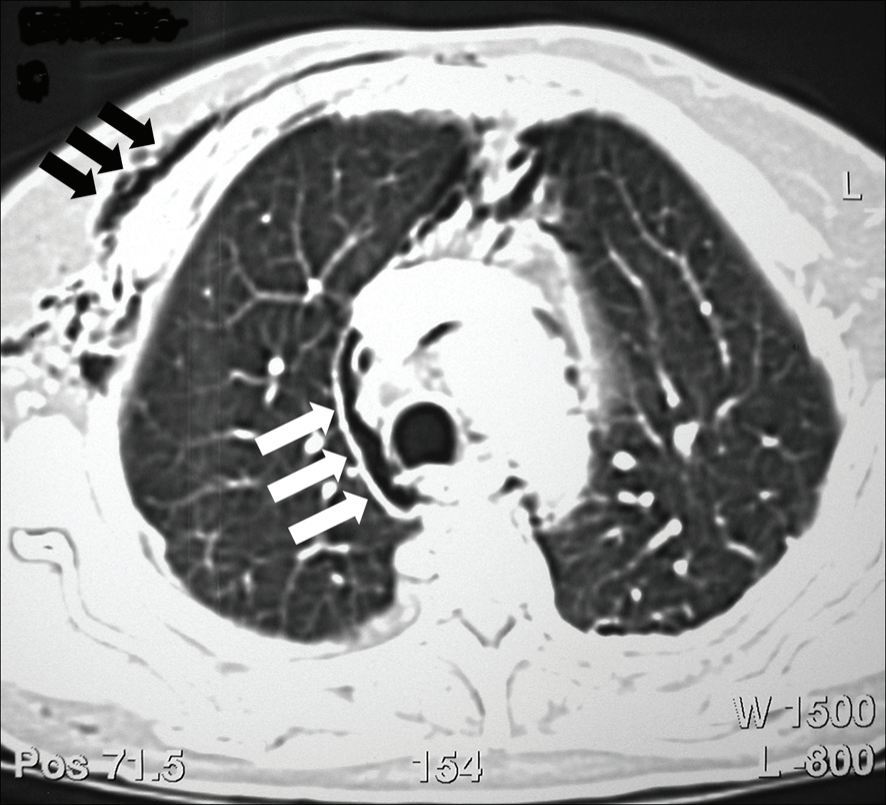
Thoracic computed tomography (CT) showing pneumomediastinum (white arrows) and subcutaneous emphysema (black arrows).

During the procedure, a peripapillary diverticula was present and a 8 mm single stone was seen in the common bile duct (Fig. 1). Bile duct diameter was 20mm. After a technically difficult sphincterotomy with a standard sphincterotome, the stone was successfully removed with standard ballon extraction technique. As soon as the procedure was finished, facial, cervical and thoracic subcutaneous emphysema were noticed. A chest x-ray revealed diffuse subcutaneous emphysema and a right-side pneumothorax (Fig. 2). These findings were confirmed by a thoracic computed tomography (CT) (Fig. 3), that also exhibited pneumomediastinum (Fig. 4). Abdominal CT was unremarkable. The patient was made NPO and intravenous administration of prophylactic metronidazole and cephtriaxone was initiated.

Twelve hours after the procedure she presented with abdominal pain and fever. At physical examination, the abdomen was soft and tender and the subcutaneous emphysema had spontaneously resolved. Laboratory test results showed leucocytosis (13,000 cells/mm^3^) without left shift and blood cultures were negative. The patient remained clinically stable and on hospital day 4 she had no fever and white blood cell count returned to normal. Plain chest x-ray was normal with no evidence of subcutaneous emphysema, pneumothorax or pneumomediastinum. The abdominal pain resolved spontaneously after seven days and dietary intake was reestablished. The patient was discharged on hospital day 10.

## Discussion

There are a few reports of pneumomediastinum, pneumothorax and subcutaneous emphysema after upper endoscopy, colonoscopy and endoscopic sphincterotomy without evidence of perforation [12-18]. Interestingly, when perforation does occur, the extension of gaseous dissection does not appear to correlate with its size [4]. During ERCP, continuous air insufflation and repeated attempts to cannulate the papilla may increase the risk of perforation due to elevated pressure over the duodenal wall [9]. When a retroperitoneal perforation occurs, the free air flows from the duodenum to the right anterior pararenal space. In this case the air may dissecate the organs inferiorly and reaches the posterior pararenal compartment. From the posterior pararenal space the air can easily reach the diaphragmatic hiatus causing pneumomediastinum, pneumothorax or cervical subcutaneous emphysema [9-19].

The therapeutic approach in cases of retroperitoneal perforation is still a matter of discussion. In a series of cases reported by Martin et al, 11 patients with perforation after ERCP were followed. Ten patients had obstructive jaundice and one had acute pancreatitis. Sphincterotomy was performed in nine cases, two of which using the pre-cut technique. The patients were treated with antibiotics, NPO and naso-gastric tube. No further therapy was necessary and the admission time range from 2 to 10 days [5].

Complete resolution with conservative management was reported in four other cases of subcutaneous emphysema following ERCP [6-8, 11]. There are two reports of retroperitoneal perforation treated surgically [9, 10], and in one of these cases the perforation site was not identified during surgery [10].

In conclusion, subcutaneous emphysema, pneumothorax and pneumomediastinum are infrequent complications of ERCP and do not appear to change the prognosis of these subjects. CT is specially helpful for diagnosis and follow-up of these patients and conservative management with NPO, naso-gastric tube placement and antibiotic therapy seems to be an appropriate first-line approach.
